# Tutorial: Saturation Transfer Difference NMR for Studying Small Molecules Interacting With Nanoparticles

**DOI:** 10.1002/mrc.70038

**Published:** 2025-09-10

**Authors:** Sekinah O. Dauda, Rajan Rai, Stephanie P. Palma, Hui Xu, Leah B. Casabianca

**Affiliations:** ^1^ Department of Chemistry Clemson University Clemson South Carolina USA

**Keywords:** ^1^H, nanoparticle, NMR, saturation transfer difference, tutorial

## Abstract

Saturation transfer difference (STD) NMR is a robust, versatile technique for detecting small molecules binding to large receptors. In addition to identifying binding molecules in the presence of nonbinding molecules, the STD‐NMR technique can be used to determine epitope maps and binding constants. In recent years, this technique has been applied to small molecules interacting with nanoparticles. In this tutorial, we introduce the technique of STD‐NMR and how it can be used to gain information about small molecules interacting with nanoparticle surfaces. After describing the principle of the STD‐NMR technique, we will explain how to best prepare the sample, set up the experiment, and analyze the resulting data when nanoparticles are involved. We will also present extensions to the STD‐NMR technique, alternative approaches for when STD‐NMR is not ideal, and future directions for the field.

## 1 Introduction

Saturation transfer difference (STD) NMR is a well‐known technique for identifying and characterizing binding between small molecule ligands and large receptors. Historically used to study small molecules interacting with protein receptors, STD‐NMR has become widely recognized as an important technique in fragment‐based drug discovery. STD‐NMR has the advantage that it is a ligand‐detected technique. Therefore, there is no requirement that the receptor be visible in solution‐state NMR. This has allowed the technique to be used with large receptors that are too large to be seen by solution‐state NMR and has sparked our interest in using STD‐NMR to examine binding between small molecule ligands and organic nanoparticles. STD‐NMR has a variety of applications, including identifying binding ligands in the presence of nonbinding molecules [1, 2], identifying the epitope on the ligand that is responsible for binding [2], determining binding constants if STD effects are measured at a variety of ligand:receptor ratios [3], and identifying competitive binding [4]. Other exciting, more exotic applications include examining monomers binding to large aggregates of the same species [5, 6, 7], determining the binding epitope of the receptor using reverse STD [8, 9, 10, 11], and even determining the charge on a surface by identifying which charged ions bind [12]. Like all NMR techniques, STD‐NMR is noninvasive and has even been performed in living cells and on cell surfaces [13, 14]. Applying STD‐NMR to study small molecules interacting with nanoparticles, therefore, has the potential to reveal novel insight into binding strength and binding epitope for different ligands, which can lead to a better understanding of the nature of the nanoparticle surface.

One of the first examples of applying the STD‐NMR technique to nanoparticles was demonstrated by the Martins group [15]. They used STD‐NMR as a noninvasive way to probe the interactions between a dispersant, SDS, and nanoparticles composed of pigment molecules. Using this technique, they observed a change in binding geometry from a flat, edge‐on binding geometry to one in which the SDS molecules form a hemicylindrical structure on the nanoparticle surface as the SDS concentration is increased. Similarly, Muñoz‐García et al. [16] used STD‐NMR spectroscopy to create an epitope map of dopamine interacting with POPG lipid nanodiscs with a hydrodynamic radius of approximately 5 nm. In contrast, negligible interactions were observed between dopamine and POPC or POPE lipid nanodiscs.

The Zimmerman group has designed single‐chain nanoparticles (SCNPs) consisting of organic polymers. When these SCNPs encapsulate metals, they can be used as catalysts. STD‐NMR was used to clearly observe interactions between the SCNPs and binding substrates, whereas no STD effect was observed between the SCNP and substrates that were not catalyzed by the SCNP [17]. The same group also used STD‐NMR to identify binding between a Ru‐SCNP and β‐galactosidase, which catalyzes an orthogonal reaction intracellularly. The small size of the SCNP (~7 nm) allowed the STD effect on the protons of the SCNP to be observed [18].

The Mancin and Rastrelli groups have used STD‐NMR combined with other techniques including nuclear Overhauser effect spectroscopy (NOESY) for NMR‐based chemosensing [19, 20]. By selectively enhancing the signals of molecules that bind to nanoparticles, this method has been used to simplify spectra and “sense” only those molecules that bind to the nanoparticles. By tuning the kind of nanoparticle used, the authors have been able to sense small molecules with desired characteristics. They have investigated the reasons behind the lower limit of detection of their technique [21].

Kundu et al. [22] used STD‐NMR to show that the drug doxorubicin was encapsulated in polymer coacervates. Above the cloud point temperature, when coacervates form, a decrease in the STD effect is observed for doxorubicin protons. This is attributed to an increase in the binding constant between the drug and polymer, leading to a decrease in *k*_off_, which leads to a decrease in the STD effect.

Our group has used STD‐NMR to study small molecules binding to the surface of plastic nanoparticles. These organic nanoparticles have a dipolar‐coupled network of proton spins, which facilitates the spread of saturation through the nanoparticle. We have used STD‐NMR to determine binding constants between polystyrene nanoparticles (PSNPs) and alcohols [23] and used it to determine the binding epitope of cyanine [24] and xanthine [25] dyes. STD‐NMR was used to identify the intermolecular forces that are responsible for binding between amino acids and the surface of functionalized PSNPs, specifically π–π interactions, electrostatic effects, and London dispersion interactions in long‐chain amino acids [26, 27]. Competition STD‐NMR was used to show that the antibiotic levofloxacin out‐competes the other antibiotics amoxicillin and metronidazole for binding sites on PSNPs [28]. In other environmental applications, we have shown that STD‐NMR can be used to show that aromatic amino acids bind less strongly to nanoparticles composed of nonaromatic plastic and that the STD‐NMR experiment can be done in natural water samples with only a small amount of D_2_O added [29]. Last, we have included STD‐NMR of small molecules interacting with plastic nanoparticles in a toolbox of techniques that can be used to probe the binding of polyfluorinated substances with nanoscale plastic to understand how these two pollutants interact with each other [30].

In this tutorial, we will introduce the principle of the STD‐NMR technique and describe the sample preparation, experiment setup, and data analysis required for STD‐NMR, with special attention paid to experiments involving small molecules interacting with nanoparticles. In the last sections, we will describe extensions to the STD‐NMR technique, alternative approaches that can give complementary information, and our thoughts regarding the future of the field.

### 1.1 Principle of the STD‐NMR Technique

As the name implies, this technique relies on the transfer of saturation from the receptor protons to the bound ligand protons [1, 2]. To construct an STD difference spectrum, two NMR experiments are performed. The first experiment is called the off‐resonance or reference spectrum, and it should be similar to the standard proton NMR spectrum. To obtain the reference spectrum, the saturation frequency is set to a value that is far from the resonance frequencies of both the ligand and the receptor protons. Saturation is performed at the same power for the same amount of time as in the on‐resonance experiment, but at this off‐resonance frequency, which should not disturb either the ligand or receptor resonances. In the second experiment, the on‐resonance experiment, a saturation frequency is chosen so that only receptor protons are selectively saturated. This may be achieved by choosing a saturation frequency near the edge of the spectrum, for example −1 ppm or 12 ppm for proton, because the receptor may often have a broad resonance instead of exhibiting sharp NMR lines. The receptor can often be saturated at these frequencies even if no signal is observed. During saturation of the receptor protons, the saturation spreads over the entire receptor molecule by spin diffusion. Some of the saturation transfers to bound ligands due to the intermolecular nuclear Overhauser effect (NOE). This saturation transfer causes a decrease in the intensity of the proton peaks of the bound ligands.

On the other hand, species that do not interact with the receptor will not receive any saturation, so there should be no change in the signal intensities of peaks belonging to noninteracting species between the on‐ and off‐resonance spectra. The difference spectrum is then obtained by subtracting the on‐resonance spectrum from the off‐resonance spectrum. Peaks belonging to binding ligands have a lower intensity in the on‐resonance spectrum than in the off‐resonance spectrum, and peaks belonging to nonbinding ligands have the same intensity in the on‐ and off‐resonance spectra. The difference spectrum therefore contains signals only from ligands that interact with the receptor surface during the saturation time. It is important to note that, in STD‐NMR experiments, observation of saturation transfer relies on the rapid exchange of ligand molecules between the bound and free states. When a ligand binds very tightly and dissociates extremely slowly (i.e., with a negligible *k*_off_), it can lead to a lack of observable STD signals. Therefore, the absence of ligand peaks in the STD difference spectrum can result from either no binding or from very strong binding with limited exchange and should not be interpreted as definitive evidence against interaction.

The STD effect [2] is a quantitative measure of the degree of saturation transfer for each NMR peak. The STD effect is defined as the intensity of the peak in the difference spectrum ( I) divided by the intensity of the same peak in the reference spectrum ( $I_{0}$).

(1) $$\mathrm{STD}\;\text{effect}=\frac{I}{I_{0}}$$

**FIGURE 1:**
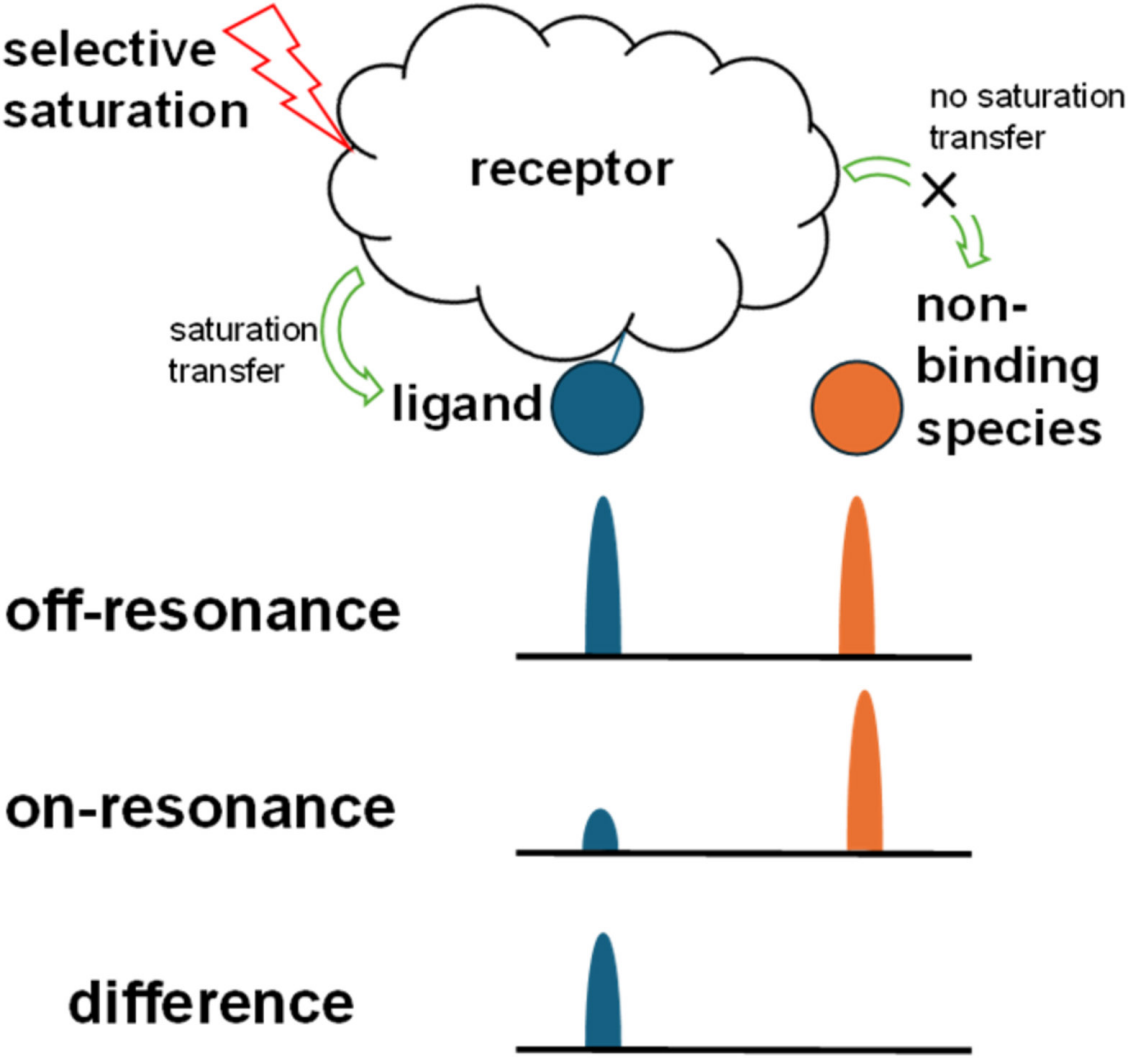
A visual representation of the saturation transfer difference NMR experiment. In the on‐resonance experiment, the receptor is selectively saturated by applying a long, weak radiofrequency pulse at a frequency at which the receptor resonates, but which will not disturb the ligand resonances. This saturation is transferred to binding ligands, leading to a decrease in signal intensity of NMR peaks corresponding to these ligands. Nonbinding species do not have an opportunity to receive any saturation transfer, so their peak intensities will be the same in the on‐ and off‐resonance spectra.

Figure 1 shows a visual representation of how the STD‐NMR experiment works.

**FIGURE 2:**
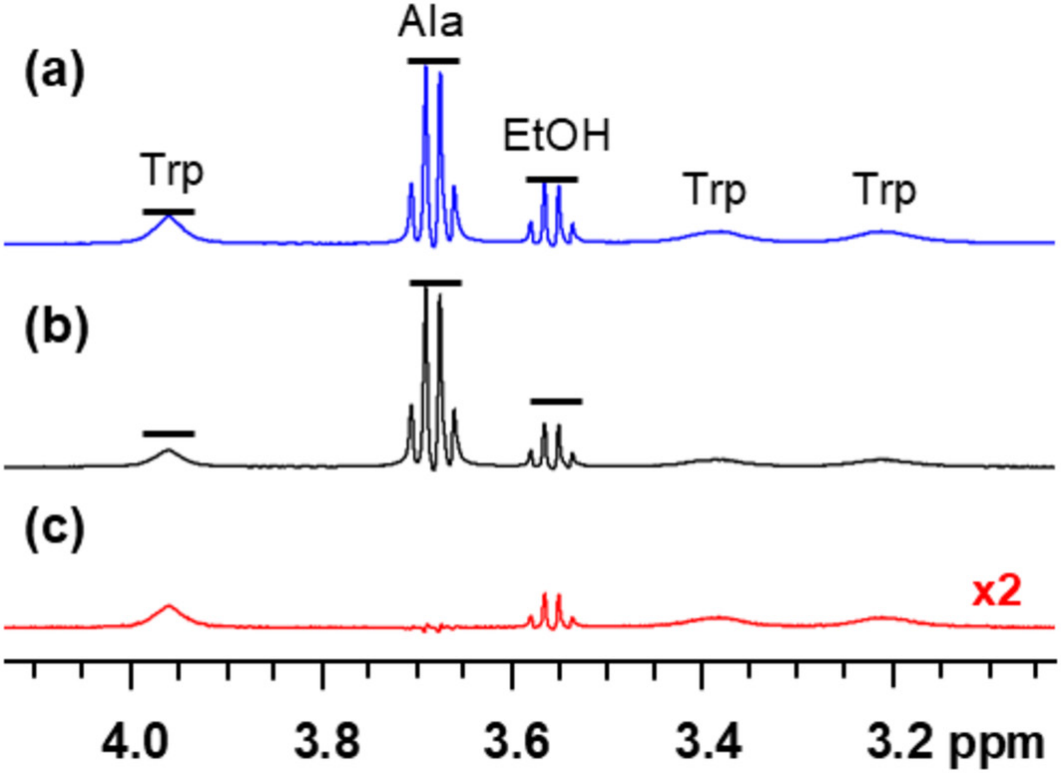
STD‐NMR experiment for a mixture of alanine (Ala), ethanol (EtOH), and tryptophan (Trp) in the presence of PSNPs: (a) off‐resonance spectrum, (b) on‐resonance spectrum, and (c) difference spectrum (×2). Alanine does not bind, so it has the same intensity in the on‐ and off‐resonance spectra, leading to no signal in the STD difference spectrum. Ethanol and tryptophan do interact with the nanoparticle surface, leading to the transfer of saturation and a decrease in signal intensity in the on‐resonance spectrum and an observable signal in the difference spectrum. For clarity, only a portion of the ^1^H NMR spectrum is shown.

A real example of off‐resonance, on‐resonance, and STD difference spectra is shown in Figure 2. This sample contained alanine, ethanol, and tryptophan in the presence of 40‐nm PSNPs. The blue spectrum is the off‐resonance spectrum, which is similar to the 1D proton NMR spectrum of this sample. Note that the tryptophan signals are broadened in the presence of the PSNP, which is already an indication of binding. In the on‐resonance spectrum shown in black, the signal intensity of the ethanol and tryptophan peaks has decreased. This decrease in signal intensity can be difficult to see, which is why the difference spectrum is important. The difference spectrum is shown in red and clearly indicates that ethanol and tryptophan exhibit an observable STD effect. The peak corresponding to alanine, which does not bind, is not observed in the difference spectrum. Only a small subtraction artifact is present.

A very clear explanation of the STD technique is given along with a nice experiment for an undergraduate laboratory in reference [31].

## 2 Sample Preparation

When using STD‐NMR to investigate the interaction between small molecules and nanoparticles, there are not many issues to consider in sample preparation. Most samples can be prepared in a straightforward manner by combining stock solutions of the small molecule analyte and the nanoparticle receptor. It is important to ensure that the small molecule ligand is fully dissolved in the solvent of interest, as aggregation can lead to false positives [5, 6, 7].

NMR is a notoriously insensitive technique. In STD‐NMR experiments, it is important to remember that the detectable STD difference intensity may be as low as 10% of the reference intensity, or even less. In this case, achieving a signal‐to‐noise ratio (S/N) of at least 10 for the STD difference intensity requires the reference intensity to have a S/N of at least 100. Consequently, the minimum ligand concentration suitable for observing STD effects is necessarily higher than the minimum concentration needed for comparable non–STD‐NMR experiments.

STD‐NMR experiments are typically done using a large excess of ligand over receptor concentration. When deciding on the ligand:receptor ratio to use, trial and error is often required, especially because the number of binding sites on each nanoparticle is not often known. As discussed below, the ligand:receptor ratio, number of binding sites on each nanoparticle, and binding strength will all influence the STD effect. If sensitivity is a problem, increasing the amount of ligand will improve the overall STD difference intensity, even though increasing the ligand:receptor ratio leads to a decrease in the STD effect (I/I_O_) [32].

When the pH needs to be controlled, using a phosphate buffer in sample preparation is not a problem. One should try to keep the buffer concentration as low as possible, because high salt concentration can disrupt electrostatic interactions between small molecules and nanoparticles, leading to a decrease in binding when electrostatic effects are the primary driving force for the interaction [27, 29]. This will lead to a lower observed STD effect. Previous work in our lab [29] has shown that when working with environmental water samples, the experiments work perfectly well in H_2_O (with water suppression, see below) with just enough D_2_O added to the sample for the purpose of locking the NMR spectrometer.

One issue that can arise regarding sample preparation for STD experiments involving nanoparticles is potential aggregation or settling of the nanoparticles. These large particles are typically suspended in solution, rather than being truly dissolved. We typically work with organic nanoparticles that have charged groups on the surface, which allows them to remain suspended in solution for up to several months with no observable sample degradation. Other nanoparticle preparations, however, may have problems with nanoparticles settling out of solution, leading to a lack of homogeneity of the sample. Egner et al. [33] present a clever solution to this problem. By using an aqueous agarose gel to stabilize the mixture, they were able to perform solution‐state NMR experiments on a nanoparticle‐containing sample without experiencing nanoparticle aggregation or sedimentation. They have also compiled a library of organogels that can be used to solubilize nanoparticle‐small molecule systems in solvents other than water [34].

## 3 Experiment Setup

### 3.1 Control Experiments

It is important to perform control experiments on samples containing only the small molecule of interest in the absence of nanoparticles, to ensure that false positives are not being observed. The most likely cause of false positives in STD‐NMR experiments is that the saturation is directly saturating the ligand resonances. Aggregation of the ligand can also lead to false positives, because the aggregates can act as receptors with broad NMR resonances and rapid spin diffusion through the aggregate. The control samples should be made with the small molecule at the same concentration as in the actual samples, and STD‐NMR experiments should be performed on these control samples using the same NMR parameters, including recycle delay, saturation frequency and power, number of scans, etc. as in the actual experiments.

**FIGURE 3:**
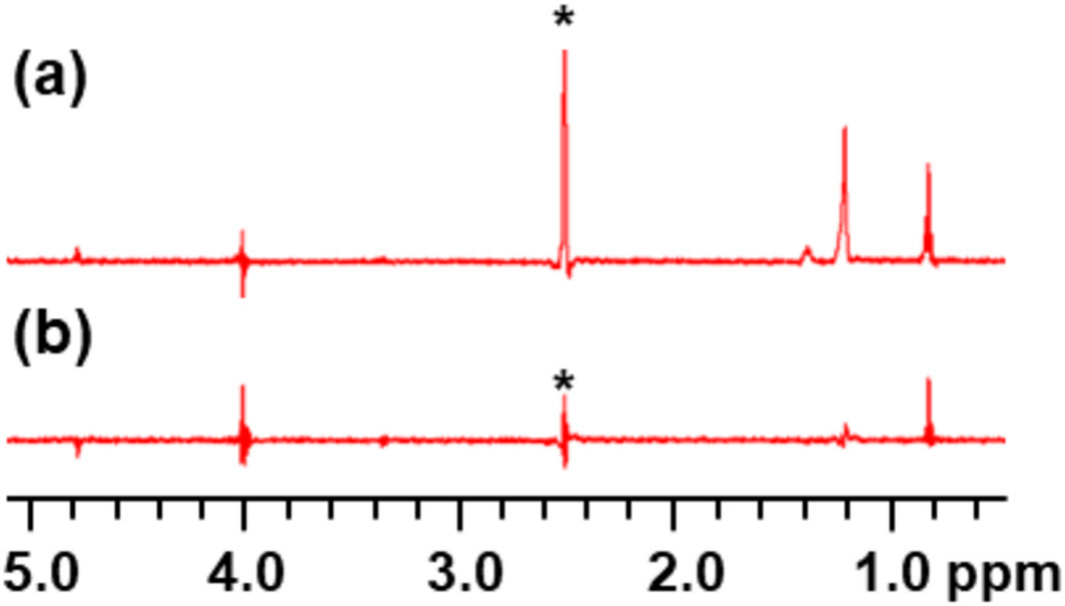
STD difference spectra from control experiments for octanol. The sample consisted of octanol dissolved in DMSO‐d6, in the absence of any nanoparticle receptor. (a) The on‐resonance saturation frequency was set to −1 ppm. (b) The on‐resonance saturation frequency was set to 12 ppm. Notice that saturating at −1 ppm results in significant signal in the STD difference spectrum, especially in the 0.5‐ to 1.5‐ppm region. * indicates residual DMSO signal.

An example of a control experiment indicating false positive STD signals is shown in Figure 3. In this figure, when −1 ppm was used for the on‐resonance saturation frequency (Figure 3a), it was observed that the small molecule was being directly saturated, as seen by residual signal in the STD difference spectrum. Once the on‐resonance frequency was changed to 12 ppm (Figure 3b), no STD effect was observed in the difference spectrum, which means that this saturation frequency is far enough away from the small molecule to be used in the actual experiments. This demonstrates the importance of carrying out a control experiment containing only the small molecule before doing the STD‐NMR experiment, to ensure that only the nanoparticle is being selectively saturated.

If STD effects are observed on the small molecule in the absence of the receptor, a different on‐resonance frequency can be used, or the saturation power can be decreased. If aggregation is the problem, a lower ligand concentration can be used.

### 3.2 How to Choose On‐ and Off‐Resonance Frequencies

The on‐resonance frequency should be chosen so that saturating at this frequency does not disrupt the ligand resonances, as demonstrated with a control experiment as described above. The off‐resonance frequency is usually chosen to be far from the typical spectral region of protons, typically 40 ppm.

**FIGURE 4:**
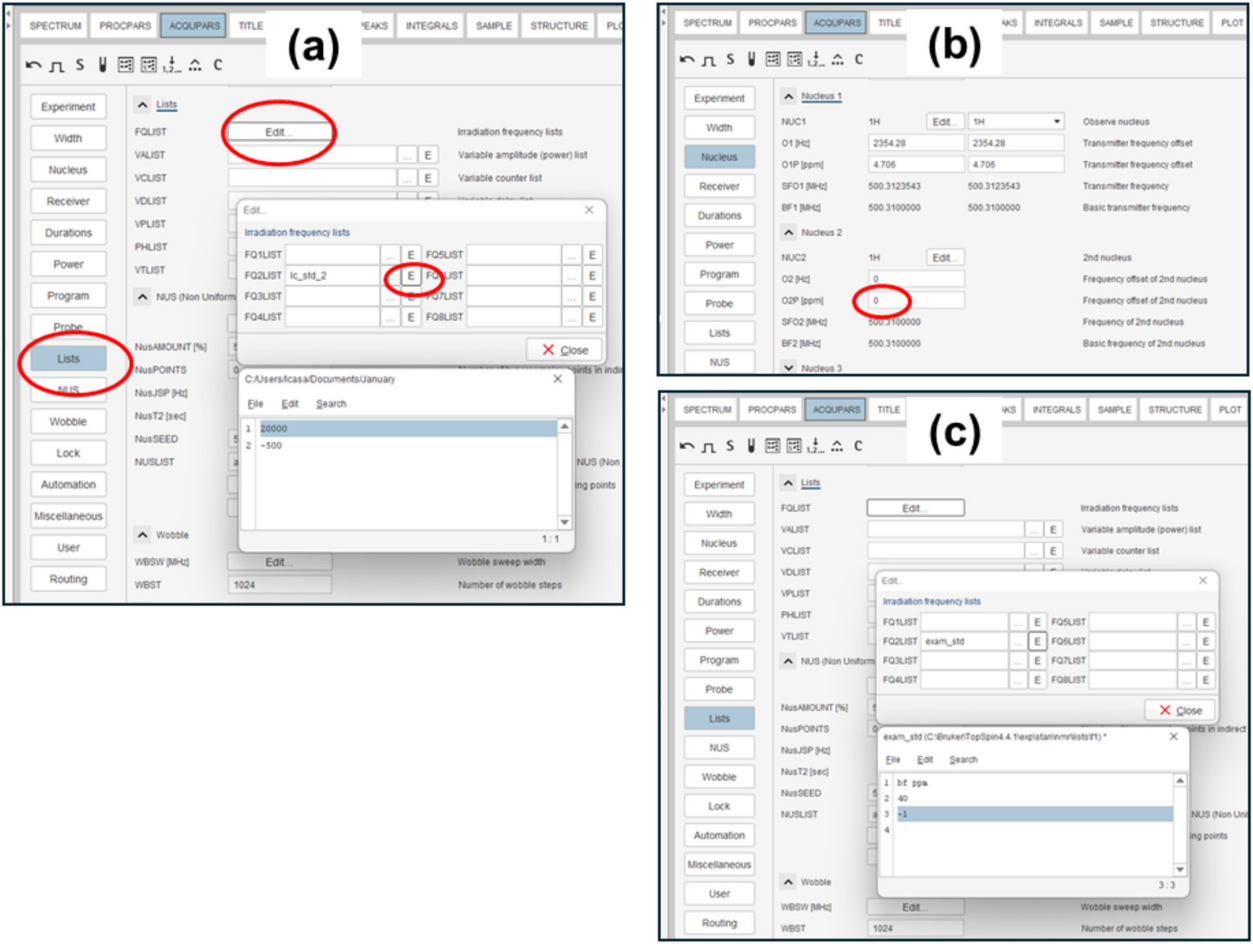
Screenshots from Topspin version 4.4.1 showing (a) how to set on‐ and off‐resonance frequencies, (b) the effect of O1p on SFO1, and (c) an alternative way to define the on‐ and off‐resonance frequencies, making use of the “bf ppm” keywords.

In Bruker, once the on‐ and off‐resonance frequencies have been decided on, these frequencies are stored in a frequency list. This list will be stored in ACQUPARS/Lists/FQLIST/FQ2LIST. The easiest way to do this is to select Lists from the Acqupars menu in Topspin, followed by clicking Edit next to FQLIST, then clicking the E or “…” to edit or change the list. This is shown in Figure 4a. We recommend creating a new list with a different name for each new combination of on‐ and off‐resonance frequencies to avoid confusion when recalling experiments in the future.

### 3.3 How to Define On‐ and Off‐Resonance Frequencies in Bruker Topspin

When setting the frequencies in Bruker's Topspin software [35], there are two options for the reference frequency and two options for the frequency units. The default is that the frequency is interpreted in Hz with respect to the base frequency SFOn. These options can be defined with the keywords “sfo Hz” or without any keywords, as shown in Figure 4a. When defining the frequencies with respect to SFO, care is needed to remember that SFO is related to the value of “O2” or “O2p” in the stddiff pulse sequence. This is shown in Figure 4b. Usually, the value of “O1” is set near 4.7 ppm or the water resonance frequency. This is the case for O1 in Figure 4b. Notice that here, SFO1 and BF1 differ by O1, which is 2354.28 Hz. If O2 is also set to 4.7 ppm, an on‐resonance frequency of −1 ppm will actually result in saturating at 3.7 ppm, since the default is to use SFO1 as the reference frequency. To avoid this confusion, since the stddiff pulse sequence uses nucleus 2 for saturation, we always make sure that O2 and O2p are set to 0 ppm. An alternative is to define the saturation frequencies with respect to the base frequency BF, which does not change when O1 or O2 changes. To confirm that this is true, compare BF1 and BF2 in Figure 4b. Figure 4c shows an example of defining the frequency list with respect to BF and in ppm units rather than Hz. The frequency list in Figure 4c contains the same frequencies as in Figure 4a, but these are now defined with respect to BF2 using the keyword bf at the top of the frequency list. The keyword ppm is also used in order to define the frequencies in ppm units, which may be more convenient than defining the frequencies in Hz.

**FIGURE 5:**
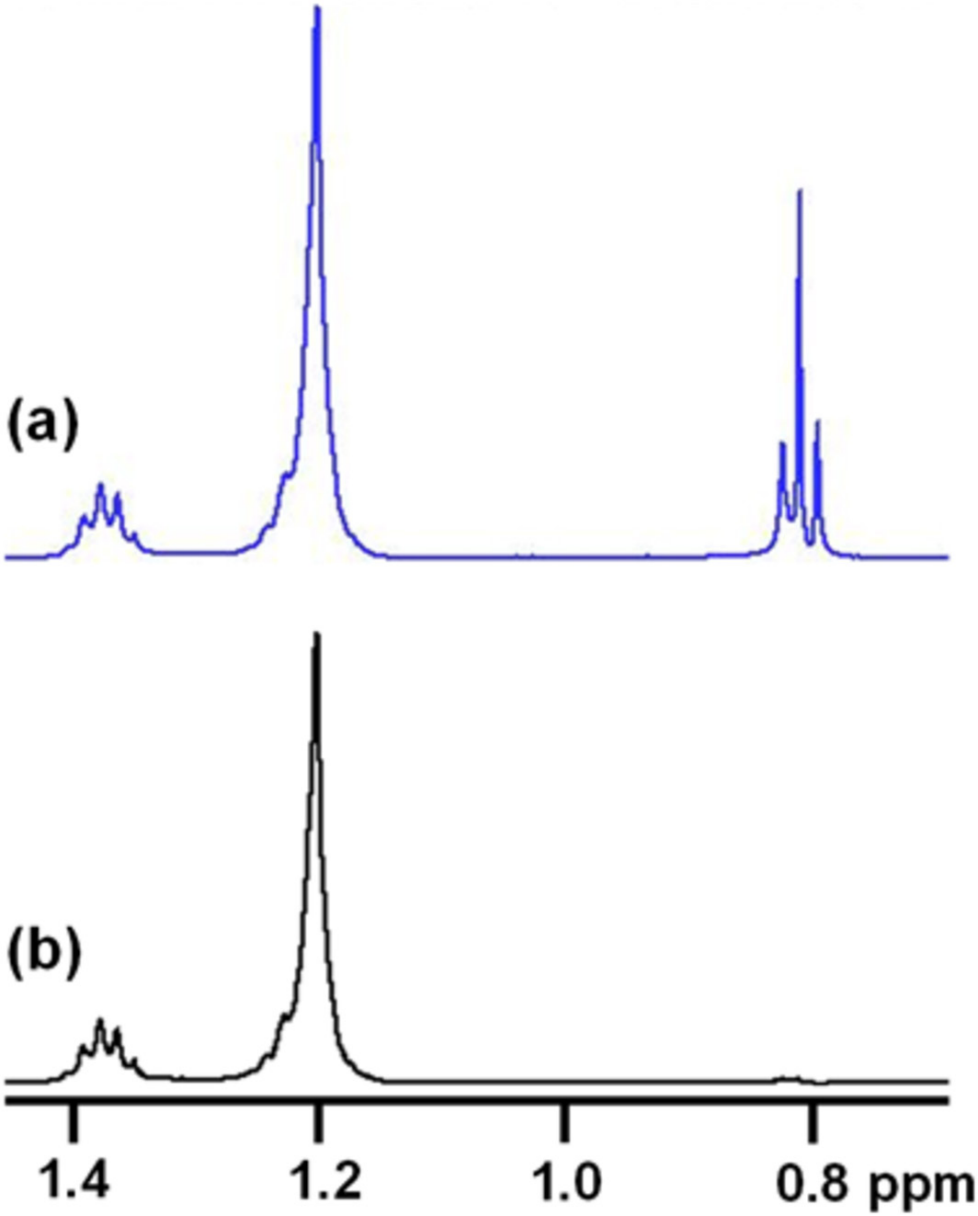
A portion of the ^1^H (a) off‐resonance and (b) on‐resonance STD spectra for octanol. In (b), the on‐resonance frequency has been set to 0.8 ppm, resulting in complete disappearance (saturation) of the octanol peak at 0.8 ppm. The disappearance of this resonance indicates that saturation is in fact occurring at 0.8 ppm.

To check that the frequency is defined correctly, it is recommended to do a test experiment in which a specific resonance of the ligand is deliberately saturated. This is shown in Figure 5, which is a portion of the off‐resonance (Figure 5a, blue) and on‐resonance (Figure 5b, black) spectra for octanol in DMSO‐d6 in the absence of any nanoparticle receptor. Here, the on‐resonance frequency was set to 0.8 ppm, exactly on resonance with the octanol CH_3_ peak. Irradiating at 0.8 ppm completely saturates this resonance, so that it disappears in the on‐resonance spectrum. Obviously, this is not an ideal on‐resonance frequency for the STD experiments, but doing this kind of test experiment is important to give the experimenter confidence that the resonance frequency is being assigned correctly.

### 3.4 Saturation Parameters

When considering how long to saturate the sample, most STD buildup curves build up within 2 s, but some require 5 or even 10 s to build up completely. When performing multiple experiments in order to create a buildup curve, the same recycle delay should be used for each experiment. In Bruker, d20 is the saturation time and d1 is the recycle delay, which includes the saturation time. In other words, if d1 is 10 s and d20 is 2 s, the time between scans will consist of 8 s without saturation followed by 2 s of saturation. Other spectrometers and software may define these times differently, so it is important to check before beginning experiments.

Saturation is usually achieved by performing a train of shaped pulses, for example Gaussian pulses, at a low power. The shape and length of the pulse may be optimized empirically for the highest STD effect. Similarly, the pulse power may be optimized as well. A higher power will excite a larger bandwidth; in other words, it will be less selective. If there are ligand peaks near the saturation frequency, a lower power may be desired, while a higher power is more likely to saturate the entire receptor. In our experience, we have found that the results are not highly sensitive to the choice of pulse power, shape, or length, but these may be important in other nanoparticle‐ligand systems.

Last, we have found in our experience that using at least 4 dummy scans before beginning acquisition is necessary to obtain reliable results.

### 3.5 Water Suppression

Often suppression of the water (or other solvent) peak may be desired in an STD‐NMR experiment in order to reduce the dynamic range of the spectrum or to better observe peaks of interest near the solvent peak. However, since the STD‐NMR experiment requires saturation prior to beginning the experiment, typical presaturation‐based sequences, such as presat [36, 37] or presaturation utilizing relaxation gradients and echoes (PURGE) [38] are less commonly used as they require a second spectrometer channel.

Instead, methods that effectively suppress water signals by some manipulation of the magnetization after the main pulse sequence should be selected. Suitable water suppression sequences include excitation sculpting [39] or water suppression by gradient tailored excitation (WATERGATE) [40, 41]. We have found excitation sculpting to be a satisfactory method for suppressing the water signal in our STD‐NMR experiments, although some attenuation of signals near the water peak is observed. As long as the degree of attenuation is the same in the reference and difference spectra, this should not influence the measured STD effect. WATERGATE has the advantage that it does not suppress exchangeable protons [42], but is slightly more complicated to set up as it requires optimizing one or more delays for binomial water suppression.

## 4 Data Processing and Analysis

The Bruker macro “stdsplit” is used to combine the collected on‐ and off‐resonance spectra into a reference and difference STD spectrum. After running “expinstall,” the stdsplit macro can be run from the command line. When running this macro, we find it convenient to save the reference spectrum in the experiment number appended with 001 and the difference spectrum in the experiment number appended with 002. In other words, if the original STD experiment is stored in experiment 4, the reference spectrum will be stored in experiment number 4001 and the difference spectrum will automatically be stored in experiment number 4002. When collecting a series of STD spectra at different saturation times in order to create an STD buildup curve, the MATLAB code included in Appendix A can be used to quickly obtain the reference and difference integrals of each peak of interest. These integrals can then be imported into Excel or similar software to create the buildup curves.

**FIGURE 6:**
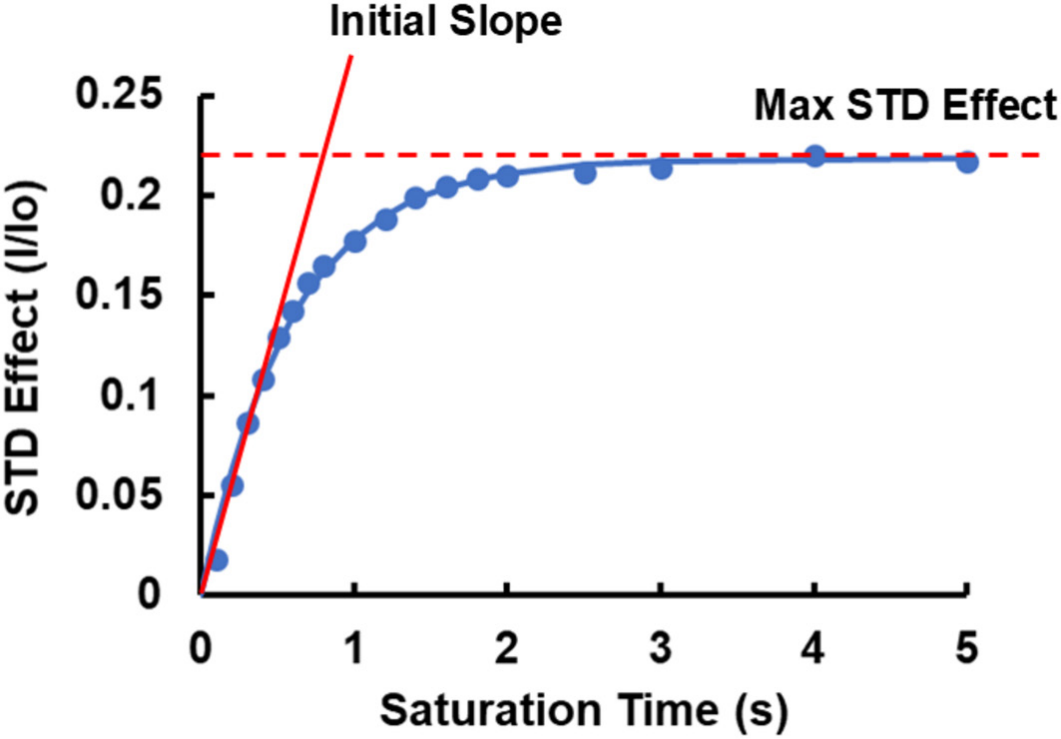
Example STD buildup curve showing the initial slope and maximum STD effect. The experimental points (blue dots) are fit by a curve (blue solid line) according to the equation $S=S_{\max}\left(1-e^{-\mathrm{kt}}\right)$.

The STD buildup curve can then be fit to the following equation (see Figure 6) to obtain the maximum STD effect (*S*_max_) and the STD buildup constant (*k*):

(2) $$S=S_{\max}\left(1-e^{-\mathrm{kt}}\right)$$

The initial slope of the buildup curve can then be calculated from:

(3) $$\frac{\partial S}{\partial t}=S_{\max}ke^{-\mathrm{kt}}$$

(4) $${\left.\frac{\partial S}{\partial t}\right|}_{t=0}=S_{\max}k$$

According to Angulo and Nieto [3], the initial slope of the STD buildup curve is a better metric to use than the STD effect at a single saturation time when calculating the dissociation constant (*K*_D_) for binding. The calculation of *K*_D_ is done by collecting a series of STD buildup curves at different ligand:receptor ratios and fitting the results to a Langmuir isotherm. Several difficulties occur when using this method to examine small molecules binding to a nanoparticle receptor rather than a protein receptor. One is that the nanoparticle surfaces may have several binding sites, whereas proteins generally have only one or two active sites. Modeling small molecule binding to nanoparticles can be done by replacing the concentration with a value that is proportional to the nanoparticle surface area for each nanoparticle [15, 23]. The *K*_A_ and number of binding sites are present in the equation as a product, so a fit will give the product of *K*_A_ and number of binding sites only. If one is known or can be assumed, the other can be determined. Other issues with nanoparticles include polydispersity and binding site heterogeneity. For example, it may even be the case that both strong and weak binding are observed simultaneously in the same nanoparticle‐ligand system [28]. Strong binding is observed as a decrease in NMR signal intensity of the ligand and often no observable STD effect, while weak binding manifests as an increase in linewidth of the ligand NMR signal (decrease in *T*_*2*_ relaxation time) and an observable STD effect. These difficulties mean that often absolute quantification of *K*_D_ and/or ligand‐receptor distances in nanoparticle‐small molecule systems is not always straightforward or even possible.

The STD effect observed depends on several factors, as described in detail by Jayalakshmi and Krishna [43, 44] in their complete relaxation and conformational exchange matrix (CORCEMA) analysis. These factors include the saturation time (which enables us to create an STD buildup curve); the distance between the receptor and the nucleus in question (the basis for STD epitope mapping); exchange lifetimes, *k*_on_ and *k*_off_, and the dissociation constant *K*_D_; the ligand:receptor ratio (the reason for defining an STD amplification factor); the rotational correlation time of the complex and free ligand; and the *T*_1_ relaxation time of each nucleus.

The dependence of the STD effect on the distance between the receptor surface and the nucleus for which the STD effect is being measured is the basis for STD epitope mapping. The transfer of saturation occurs through the NOE, which has a well‐known 1/r^6^ distance dependence, where r is the distance between the two nuclei. Therefore, nuclei that are located closer to the receptor surface in the bound complex will receive more saturation transfer and exhibit a higher STD effect. The strong dependence on r has been exploited in many studies in order to obtain atomic‐level structural models of ligands in the bound configuration [45, 46, 47, 48, 49, 50, 51]. However, the ligand‐receptor distance is not the only factor that influences the STD effect, and these other factors must be kept in mind when designing STD‐NMR experiments to determine distances, binding constants, or structures of the bound complex. Many of these factors can be controlled by the experimenter. For example, knowing that the STD effect is dependent on saturation time and ligand:receptor ratio, when comparing STD effects for two different small molecules interacting with the same receptor, the concentrations and saturation times used can be kept the same. When comparing STD effects between nuclei on the same ligand interacting with the same receptor, the rotational correlation times of the complex and free ligand, exchange lifetimes, and dissociation constants will be the same.

The STD effect will depend on the ligand:receptor ratio. If the concentration of receptor is kept constant, increasing the concentration of ligand will increase the amount of ligand that is able to bind to the receptor, receive transfer of saturation, and dissociate from the receptor during the saturation time. However, as the total amount of ligand is increased, the amount of bulk or free ligand also increases, and this also increases the amount of ligand that is not saturated during the saturation time. This bulk ligand that is not saturated contributes to the reference intensity. Hence, although the STD difference intensity increases with increasing ligand:receptor ratio, the STD reference intensity also increases due to the bulk ligand that does not receive the saturation transfer. The increase in the reference intensity is larger, so increasing the ligand:receptor ratio leads to a decrease in the STD effect [32]. Another way to think about it is that increasing the amount of receptor while keeping the amount of ligand constant leads to an increase in the fraction of bound ligand. Again, increasing the ligand:receptor ratio leads to a decrease in the STD effect. A larger ligand:receptor ratio also decreases the probability of ligand rebinding. Rebinding decreases the overall STD effect, since ligands that receive saturation transfer, dissociate from the receptor, then rebind do not contribute to the overall number of ligands that have been saturated and are free in solution [3].

The influence of ligand:receptor ratio on the STD effect can be normalized by introducing the STD amplification factor, STD_AF:

(5) $$\mathrm{STD}\_\mathrm{AF}=\mathrm{STD}\;\text{effect}\times \frac{\left[L\right]}{\left[R\right]}$$

**FIGURE 7:**
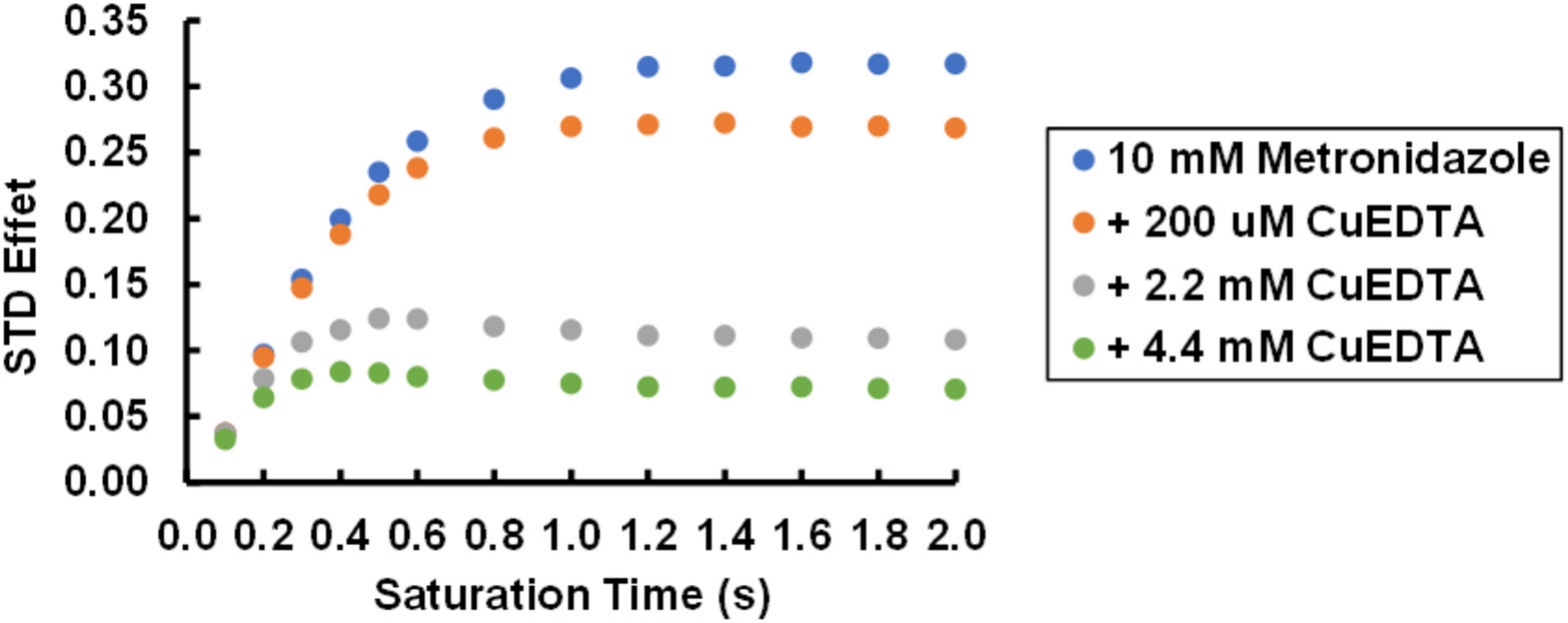
Effect of *T*_1_ relaxation time on STD buildup curve. As increasing concentrations of paramagnetic CuEDTA are added to a sample of metronidazole and polystyrene nanoparticles, the *T*_1_ of the metronidazole proton decreases. This increase in nuclear relaxation rate drives the proton magnetization back to equilibrium more quickly, leading to a lower maximum STD effect. However, this maximum STD effect is reached more quickly, such that the initial slope of the buildup curve is unaffected by the difference in nuclear *T*_1_ relaxation time.

The *T*_1_ relaxation times of each nucleus also influence the STD effect. *T*_1_ relaxation times are one factor that the experimenter cannot control when setting up STD experiments, but the *T*_1_ relaxation times can be measured. A shorter *T*_1_ relaxation time leads to magnetization being driven more quickly back to equilibrium. This causes the STD buildup curve to result in a lower STD maximum, but this maximum is reached more quickly. The combination of a higher maximum STD effect and shorter *k* leads to the initial slope of the STD buildup curve (Equation 4) remaining unchanged. This effect is demonstrated in Figure 7. This figure shows STD buildup curves for the drug metronidazole interacting with PSNPs in the presence of increasing amounts of CuEDTA. The paramagnetic copper reduces the *T*_1_ relaxation time of the metronidazole ^1^H nuclei, leading to STD buildup curves with lower maxima but faster buildup times. However, as can be seen from the figure, the initial slope of the buildup curve is not affected by the change in nuclear *T*_1_. This is to be expected, since the initial slope is found at a saturation time of zero, at which time the relaxation of the nucleus should not have time to influence the STD effect. This is another reason why using the initial slope of the STD buildup curve instead of an STD effect at one particular saturation time in analysis such as determining the *K*_D_ from a Langmuir isotherm is advantageous [3, 15].

The dependence of the STD effect on *k*_on_, *k*_off_, and correlation times of the free ligand and receptor is more complicated but is modeled in reference [44]. One disadvantage of the STD‐NMR approach is that it cannot be used to observe very strong binding, because some nonzero *k*_off_ rate is required for saturated ligand to leave the receptor surface and be observed in solution. Therefore, stronger binding can often lead to lower STD effects. It is estimated that STD‐NMR can be used to study systems with binding constants (*K*_D_) between 10 mM and 1 nM [32]. Another disadvantage of the technique is that it requires that the nanoparticle or receptor have a strong dipolar‐coupled network of protons so that after a portion of the receptor is saturated, the saturation can transfer easily to the entire receptor. Thus, STD‐NMR is not an appropriate technique to study binding between small molecules and inorganic nanoparticles, for example. However, in favorable circumstances, the CORCEMA analysis can be used to determine a detailed structure of the bound ligand [45, 46, 47, 48, 49, 50, 51].

## 5 Variations on the STD‐NMR Technique

Since the STD‐NMR concept relies on selectively saturating the receptor prior to the standard NMR experiment, the STD technique can be easily expanded to various other NMR experiments. This is done by simply prepending the alternating on‐ and off‐resonance saturation to any NMR pulse sequence. For example, two‐dimensional experiments including STD‐NOESY, STD‐TOCSY [52, 53, 54, 55, 56], and STD‐HSQC [56, 57] have been extensively used and are included in Bruker's standard pulse sequence collection.

One‐dimensional heteronuclear STD‐NMR experiments can be used to saturate ^1^H and then observe the saturation transfer on some other nucleus [8, 58, 59, 60]. This has the advantage of avoiding spectral overlap, since nuclei such as ^19^F or ^13^C have a much larger chemical shift dispersion than ^1^H. Heteronuclear STD‐NMR can also be used to probe interactions between a large receptor and specific ^19^F sites on a ligand. Simpson and coworkers [8, 9, 10, 11] have also developed reverse‐heteronuclear STD‐NMR, in which the ^19^F nucleus is saturated and the ^1^H spectrum is observed. This technique was used to identify sites in soil organic matter that preferentially interact with fluorinated organic contaminants [10].

Several methods have been devised to speed up or simplify the data collection and analysis of STD‐NMR spectra. Rocha et al. [61] have developed the reduced data set STD‐NMR (rd–STD‐NMR) approach. Instead of collecting the full buildup curve, the rd–STD‐NMR approach uses only two saturation times to approximate the initial slope of the STD buildup curve. This approximate initial slope can then be used in epitope mapping, a Langmuir isotherm to determine the binding constant, or other analyses. Collecting only two data points instead of the full buildup curve drastically decreases the time needed for data collection. Similarly, Monaco et al. [62] have combined chemical shift imaging STD‐NMR with a concentration gradient in order to collect STD buildup curves at various ligand:receptor ratios in the same sample.

STD‐NMR has been combined with high‐resolution magic angle spinning (HRMAS) to study ligands interacting with membrane proteins [63]. The HRMAS approach improves the resolution of the ligand peaks by removing the magnetic field inhomogeneity due to magnetic susceptibility differences due to the semisolid nature of these samples. Because STD‐NMR is a ligand‐detected technique, this is particularly helpful. Combining HRMAS with STD‐NMR simplifies sample preparation and allows experiments to be carried out in the natural membrane environment [63, 64]. It can identify binding ligands with low affinity that might be missed when carrying out STD‐NMR in solution [64]. The combination of HRMAS and STD‐NMR can also be used to observe changes in both the small molecule and membrane during interaction and can potentially be used for fragment‐based discovery for drugs targeting membrane proteins [63]. This HRMAS‐STD‐NMR technique has great potential to be applied to STD‐NMR of small molecules interacting with nanoparticles in the future.

## 6 Alternatives to STD‐NMR

As discussed above, although STD‐NMR is a simple, versatile technique to examine binding between small molecules and large receptors including nanoparticles, there are some systems for which STD‐NMR is not appropriate. Notably, STD‐NMR cannot be used for strongly binding systems or inorganic nanoparticles. In these systems, trNOESY, waterLOGSY, and DEST techniques can be used instead.

Two‐dimensional NOESY NMR is generally thought of as a technique that can be used to determine distances between nuclei. However, the transferred NOESY (trNOESY) technique has been used to identify molecules that bind to large receptors, including nanoparticles [65, 66, 67, 68, 69, 70, 71, 72]. Like STD‐NMR, trNOESY is a ligand‐detected technique. trNOESY is based on the opposite phase of the NOE for molecules with fast rotational correlation times, such as free small molecules in solution, compared to those with long rotational correlation times, including large molecules or bound small molecules. If the diagonal peaks of a NOESY spectrum are phased positive, ligands that bind will have strong, positive cross peaks, while ligands that do not bind will have small negative cross peaks. trNOESY has the advantage over STD‐NMR that it can be used to detect small molecules binding to inorganic nanoparticles or other receptors without a dipolar coupled network of protons. Additionally, it can be used for ligands that do not contain protons; our group has recently demonstrated the use of ^19^F–^19^F trNOESY to detect perfluorinated compounds binding to PSNPs [30].

Water‐ligand observed via gradient spectroscopy (waterLOGSY) [73, 74, 75] is a method similar to STD‐NMR that can be used to identify small molecules interacting with nanoparticles. WaterLOGSY relies on saturating bulk water; this saturation is then transferred to water molecules that are immobilized as part of the ligand‐receptor complex and finally to the bound receptor. This results in opposite phase in a 1D spectrum for molecules that are bound versus unbound. WaterLOGSY is most often used as a qualitative technique, giving a yes or no answer to whether a molecule binds or not, although it can be used for epitope mapping as well [76]. Suzuki et al. [77] used the epitope‐identifying capability of waterLOGSY to identify the residues in the Ti‐binding peptide (RKLPDA) that are responsible for the interactions of this peptide with TiO_2_ nanoparticles. Because the density of protons on the nanoparticle was small, the water peak was selectively saturated, and “STD effects” were measured, although the experiment was essentially a waterLOGSY experiment. The positively charged groups of the N‐terminal arginine and lysine residues exhibited large STD effects, indicating that these groups are responsible for binding to the negatively charged surface of the TiO_2_ nanoparticles. The same group also used waterLOGSY to show that this peptide also binds to SiO_2_ nanoparticles but that the negatively charged aspartic acid is responsible for binding to the positively charged SiOH_2_+ groups on the surface of this nanoparticle [78]. Because it relies on saturation of bulk water, waterLOGSY suffers from a lack of sensitivity in highly deuterated samples. However, it is a robust and rapid method that can quickly be used to identify binding ligands.

Dark‐state exchange saturation transfer (DEST) [79, 80, 81] is a method that is similar to STD‐NMR in that it relies on saturating a slowly tumbling species (the invisible or “dark” state) and observing the decrease in NMR signal intensity that results from the transfer of that saturation to a free small molecule. The difference between DEST and STD‐NMR is that in DEST, the bound small molecule is directly saturated, rather than saturating the receptor and relying on NOEs to transfer that saturation to the bound small molecule. Due to this difference, one advantage of DEST over STD‐NMR is that DEST can be used for large molecules and nanoparticles that are nonprotonated, since there is no requirement that the receptor be saturated [33, 82]. Site‐specific transverse relaxation rates and therefore information about dynamics of the small molecule in the bound state and the kinetics of exchange can be obtained from modeling DEST saturation profiles [79, 81].

## 7 Conclusions and Outlook

As we hope this tutorial has made clear, STD‐NMR is a simple yet elegant technique for studying interactions between small molecules and nanoparticle surfaces. STD‐NMR is easy to set up and is amenable to most of the sample preparations used in typical solution‐state NMR experiments. It can be used to determine binding constants, binding epitopes, and even structures of molecules in the bound configuration. The STD technique can be extended to 2D experiments and can be used for heteronuclear saturation transfer. Disadvantages of the technique include the requirement for the nanoparticle to have protons or another dipolar‐coupled network of spins for saturation to extend to the entire receptor. There are also both upper and lower limits on the binding constants that can be observed using this technique. Despite these limitations, we believe that STD‐NMR has a strong future in the area of small molecule‐nanoparticle interactions, especially in combination with other techniques such as trNOESY, waterLOGSY, and DEST. STD‐NMR experiments with a variety of small molecules are poised to answer important questions about the nature of the nanoparticle surface, including the number of binding sites on the nanoparticle. The combination of STD‐NMR with HRMAS in particular is predicted to be especially important in the coming years.

## Data Availability

The data that support the findings of this study are available from the corresponding author upon reasonable request.

## Appendix A

How to use this code: To create an STD buildup curve, STD experiments with the same d1 and different d20 saturation times should be collected in different experiment numbers (expno), for example in expno 3–10. After acquisition, the macro “stdsplit” should be called on each dataset, and the reference spectrum should be stored in that expno followed by “001.” The difference spectrum will then automatically be saved as the expno followed by “002.” For example, if the STD experiment is stored in experiment 4, the reference spectrum will be saved in experiment 4001 and the difference spectrum in experiment 4002. The two MATLAB scripts below, process_std.m and bruker_plot_std.m, should be stored in the same folder on the computer with the NMR data. In the file process_std.m, change ‘filename’ to your filename and the computer path to the folder or directory where your NMR data are stored, and add or delete additional lines for the experiment numbers you used. In the example, experiments 3–10 are used. Change the lines beginning with reference_integrals and difference_integrals to reflect the experiment numbers you used. On a Mac, you may also have to find and replace the backslash (\) to forward slash (/) in both Matlab scripts.

Once you have modified the process_std.m script, run this script. If there are no errors, a figure should display with all the reference spectra in blue and all the difference spectra in red. Then, for each peak of interest, record the minimum and maximum ppm for the peak integral. Input these values into iso1_min, iso1_max, iso2_min, etc. If you want to integrate more or fewer than three peaks, comment or uncomment an appropriate number of the lines beginning with isoX_ and uXindex as shown in the example. Finally, the last line should list isoX_integral where X ranges from 1 to the number of peaks, separated by a space.

When the integral limits have been input into bruker_plot_std.m, rerun process_std.m. The reference integrals are now stored in the parameter “results.” The results may be exported to a delimited text file called “myfilename.txt” by typing dlmwrite(‘myfilename.txt’,results) in the MATLAB command line.

Note that these scripts approximate the integral by summing the intensity over all points between the chosen integration limits. Therefore, they should not be used to calculate and compare integrals between spectra with different digital resolution. However, in the STD experiment, the reference and difference spectra are collected with the same parameters, including number of points and spectral width, so these scripts can be used to calculate an STD effect.

process_std.m:

offset = 4.7–8.188;

dc = 0;

figure;

ans3r = bruker_plot_std(‘filename’,3001,’C:\Users\Leah\Documents\February 2025′,offset,’b’,dc);

hold on; ans4r = bruker_plot_std(‘filename’,4001,’C:\Users\Leah\Documents\February 2025′,offset,’b’,dc);

hold on; ans5r = bruker_plot_std(‘filename’,5001,’C:\Users\Leah\Documents\February 2025′,offset,’b’,dc);

hold on; ans6r = bruker_plot_std(‘filename’,6001,’C:\Users\Leah\Documents\February 2025′,offset,’b’,dc);

hold on; ans7r = bruker_plot_std(‘filename’,7001,’C:\Users\Leah\Documents\February 2025′,offset,’b’,dc);

hold on; ans8r = bruker_plot_std(‘filename’,8001,’C:\Users\Leah\Documents\February 2025′,offset,’b’,dc);

hold on; ans9r = bruker_plot_std(‘filename’,9001,’C:\Users\Leah\Documents\February 2025′,offset,’b’,dc);

hold on; ans10r = bruker_plot_std(‘filename’,10,001,’C:\Users\Leah\Documents\February 2025′,offset,’b’,dc);

hold on; ans3d = bruker_plot_std(‘filename’,3002,’C:\Users\Leah\Documents\February 2025′,offset,’r’,dc);

hold on; ans4d = bruker_plot_std(‘filename’,4002,’C:\Users\Leah\Documents\February 2025′,offset,’r’,dc);

hold on; ans5d = bruker_plot_std(‘filename’,5002,’C:\Users\Leah\Documents\February 2025′,offset,’r’,dc);

hold on; ans6d = bruker_plot_std(‘filename’,6002,’C:\Users\Leah\Documents\February 2025′,offset,’r’,dc);

hold on; ans7d = bruker_plot_std(‘filename’,7002,’C:\Users\Leah\Documents\February 2025′,offset,’r’,dc);

hold on; ans8d = bruker_plot_std(‘filename’,8002,’C:\Users\Leah\Documents\February 2025′,offset,’r’,dc);

hold on; ans9d = bruker_plot_std(‘filename’,9002,’C:\Users\Leah\Documents\February 2025′,offset,’r’,dc);

hold on; ans10d = bruker_plot_std(‘filename’,10,002,’C:\Users\Leah\Documents\February 2025′,offset,’r’,dc);

reference_integrals = [ans3r; ans4r; ans5r; ans6r; ans7r; ans8r; ans9r; ans10r];

difference_integrals = [ans3d; ans4d; ans5d; ans6d; ans7d; ans8d; ans9d; ans10d];

results = [reference_integrals,difference_integrals];

bruker_plot_std.m:

function [plot_ans] = bruker_plot_std(filename, expno, computer_path, offset,line_color, dc).

%% read Bruker Data.

path = [computer_path ‘\’ filename ‘\’ num2str(expno)];

fullname = [path ‘\pdata\1\1r’];

bigendian = 0; % Topspin 2.x and later.

%bigendian = 1; % Topspin 1.x.

if (bigendian).

x = fopen (fullname,’r’,’b’);

else.

x = fopen (fullname,’r’,’l’);

end

[DATA_tmp,DATA_s] = fread(x,’int32’);

DATA = DATA_tmp;

fclose(x);

%% Get acq param.

acqu_par = fopen([path ‘\acqu’]);

pars = textscan (acqu_par,’%s %s');

par_names = pars{1};

par = pars{2};

np = str2double(par{find (ismember (par_names, ‘##$TD = ‘)==1)});

np = np/2;

o1 = str2double(par{find (ismember (par_names, ‘##$O1 = ‘)==1)});

o2 = str2double(par{find (ismember (par_names, ‘##$O2 = ‘)==1)});

sfo1 = str2double(par{find (ismember (par_names, ‘##$SFO1 = ‘)==1)});

sfo2 = str2double(par{find (ismember (par_names, ‘##$SFO2 = ‘)==1)});

sfrq = sfo1 + o1/100000;

sfrq2 = sfo2 + o2/100000;

sw = str2double(par{find (ismember (par_names, ‘##$SW_h = ‘)==1)});

dw = 1/sw;

fclose (acqu_par);

%% Get procs param.

proc_par = fopen([path ‘\pdata\1\procs’]);

ppars = textscan (proc_par,’%s %s');

ppar_names = ppars{1};

ppar = ppars{2};

nc_proc = str2double(ppar{find (ismember (ppar_names, ‘##$NC_proc = ‘)==1)});

fclose (proc_par);

%Display Data.

swp = sw/sfrq;

sw_inc = swp/length (DATA);

axisppm = [length (DATA):‐1:1];

axisppm = axisppm*sw_inc;

axisppm = axisppm+offset;

scale_factor = 2^(−1*nc_proc);

DATA = real (DATA)/scale_factor+dc;

plot (axisppm, DATA,line_color,’linewidth’,1);

set (gca,’XDir’,’reverse’);

experiment = [filename “num2str(expno)].

iso1_min = 1.45;

iso1_max = 1.7;

iso2_min = 3.36;

iso2_max = 3.5;

iso3_min = 8.0;

iso3_max = 8.15;

% iso4_min = 2.71;

% iso4_max = 3.02;

%

% iso5_min = 3.6;

% iso5_max = 3.8;

%

% iso6_min = 7.28;

% iso6_max = 7.35;

%

% iso7_min = 8.7;

% iso7_max = 8.86;

u1index_min = find (axisppm<iso1_min,1);

u1index_max = find (axisppm>iso1_max,1,’last’);

iso1_integral = sum (DATA(u1index_max:u1index_min));

u2index_min = find (axisppm<iso2_min,1);

u2index_max = find (axisppm>iso2_max,1,’last’);

iso2_integral = sum (DATA(u2index_max:u2index_min));

u3index_min = find (axisppm<iso3_min,1);

u3index_max = find (axisppm>iso3_max,1,’last’);

iso3_integral = sum (DATA(u3index_max:u3index_min));

% u4index_min = find (axisppm<iso4_min,1);

% u4index_max = find (axisppm>iso4_max,1,’last’);

% iso4_integral = sum (DATA(u4index_max:u4index_min));

%

% u5index_min = find (axisppm<iso5_min,1);

% u5index_max = find (axisppm>iso5_max,1,’last’);

% iso5_integral = sum (DATA(u5index_max:u5index_min));

%

% u6index_min = find (axisppm<iso6_min,1);

% u6index_max = find (axisppm>iso6_max,1,’last’);

% iso6_integral = sum (DATA(u6index_max:u6index_min));

%

% u7index_min = find (axisppm<iso7_min,1);

% u7index_max = find (axisppm>iso7_max,1,’last’);

% iso7_integral = sum (DATA(u7index_max:u7index_min));

plot_ans = [iso1_integral iso2_integral iso3_integral];

end
